# Gene expression profiling of ovarian carcinomas and prognostic analysis of outcome

**DOI:** 10.1186/s13048-015-0176-9

**Published:** 2015-07-31

**Authors:** Sheng-Yun Cai, Tian Yang, Yu Chen, Jing-Wen Wang, Li Li, Ming-Juan Xu

**Affiliations:** Department of Obstetrics & Gynecology, Changhai Hospital, Second Military Medical University, No.168, Changhai Road, Shanghai, 200433 China; Department of Hepatic Surgery, Eastern Hepatobiliary Surgery Hospital, Second Military Medical University, No.225, Changhai Road, Shanghai, 200438 China

**Keywords:** Ovarian cancer, Gene expression profile, Genetic prognostic pattern, Candidate biomarkers

## Abstract

**Background:**

Ovarian cancer (OCA), the fifth leading deaths cancer to women, is famous for its low survival rate in epithelial ovarian cancer cases, which is very complicated and hard to be diagnosed from asymptomatic nature in the early stage. Thus, it is urgent to develop an effective genetic prognostic strategy.

**Methods:**

Current study using the Database for Annotation, Visualization and Integrated Discovery tool for the generation and analysis of quantitative gene expression profiles; all the annotated gene and biochemical pathway membership realized according to shared categorical data from Pathway and Kyoto Encyclopedia of Genes and Genomes; correlation networks based on current gene screening actualize by Weighted correlation network analysis to identify therapeutic targets gene and candidate bio-markers.

**Results:**

3095 differentially expressed genes were collected from genome expression profiles of OCA patients (n = 53, 35 advanced, 8 early and 10 normal). By pathway enrichment, most genes showed contribution to cell cycle and chromosome maintenance.1073 differentially expression genes involved in the 4 dominant network modules are further generated for prognostic pattern establish, we divided a dataset with random OCA cases (n = 80) into 3 groups efficiently (p = 0.0323, 95 % CIs in Kaplan-Meier). Finally, 6 prognosis related genes were selected out by COX regression analysis, TFCP2L1 related to cancer-stem cell, probably contributes to chemotherapy efficiency.

**Conclusions:**

Our study presents an integrated original model of the differentially expression genes related to ovarian cancer progressing, providing the identification of genes relevant for its pathological physiology which can potentially be new clinical markers.

**Electronic supplementary material:**

The online version of this article (doi:10.1186/s13048-015-0176-9) contains supplementary material, which is available to authorized users.

## Background

As the fifth leading cause of cancer related to deaths, ovarian cancer just have only 30–40 % with a five year survival rate in women [1]. In American, there were 219,800 new cases and around 142,700 women succumb to this fatal disease in 2014 [2], and the lifetime risk of epithelial ovarian cancer is one of 72 women [3], what is much more worse in developing countries [4, 5]. Ovarian tumors can be classified into epithelial (60 %), germ cell (30 %) and sex-cord stromal tumors (8 %) [6], among which the vast majority of malignant ovarian cancers (80 %-85 %) [7] began in the ovarian epithelium. Unfortunately, ovarian cancer is highly asymptomatic at early stages, the epithelial ovarian cancer (EOC) is hard to be diagnosed due to the multitude of clinical and histopathological aspects [8], lack of precursor lesions [9] and their evolution [10], thus most patients with EOC are diagnosed at advanced stage and have a poor prognosis. It is reported that only 30 % in stage I or II could be cured by surgery with five-year survival rate of 90 %, in contrast, EOC in stages III or IV could spread throughout widely with 5-year survival of less than 30 % [11, 12]. Besides, malignant ovarian germ cell tumor is hard to be distinguished by medical image and always happen to young women below the age of 20.

Recently, more and more reports declared that ovarian cancer has home history, about 10 % of EOC cases are related to inherited genes like BCRA1and BCRA2 [13, 14], and ovarian germ cell tumors can be cancerous or non-cancerous tumors depend on genome difference [15–17]. Actually, cancer is a disease single of genomes or networks of molecular interaction and control, advanced ovarian cancer with a high relapse rate related to the acquirement of chemo resistance, due to it’s ability to converting the tumor cells back into a stem cell-like state. Luckily, several existing drugs [18, 19, 14] can attack the pathway and reverse the cellular transformation, thus ‘re-sensitizing’ the tumor to treatment. For these reasons, it is urgently to develop effective strategies to stratify early and advance stage patients.

Correlation networks are increasingly being used in bioinformatics applications like generating modules (clusters) of highly correlated genes, summarizing such modules using an intra-modular hub gene or the eigengene, and analysis of modules’ networks or calculating module membership measures, which can be used to identify candidate biomarkers or therapeutic targets. Currently, we use weighted correlation network analysis (WGCNA) to correlate networks facilitate network based standardized and screened gene, aim at establish an a feasible genetic method to prognostic of outcome of individual’s ovarian carcinoma, especially the bottleneck problem of epithelial ovarian cancer and malignant ovarian germ cell cancer, therefore, making an advantage to choose the most suitable chemotherapy for a certain patient.

## Materials

As the paper did not involve any human or animal study, there was no need for any ethical approval.

### Literature selecting and building

For analysis of differential genome-wide expression between patients in different cancer stages, we selected GSE12470 dataset [20], including gene array data from 35 advanced ovarian cancer patients, 8 early ovarian cancer patients and 10 non-cancer persons.

For prognostic analysis on different types of ovarian cancer, GSE14764 data set [21] was selected, which includes genome-wide expression data from 80 ovarian cancer patients. In addition, GSE63885 dataset [22] and GSE49997 dataset [23] were tested to verify the established prognostic analysis model, these two data sets are consisted of genome-wide expression data from 101 candidates with differential ovarian cancer and mRNA expression data from 204 candidates suffered from ovarian cancer respectively.

### Database search

Gene Expression Omnibus [24] functional genomics repository was searched for the relationships between the probe in the platforms used in the selected datasets and corresponding genes. One probe set (contain several probes, N ≥1) matching one target gene, therefore average value [25] of different corresponding probe IDs is represent one gene expression level. Skew distribution of gene expression was transformed to skew normal distribution by log2 transformed and final probe set level data was generated through Robust Multichip Analysis [26] (a model-based algorithms) with default parameters [27].

### Screening of differentially expressed genes

After expression data for post-processing of standardization, we directly employed a more mature significance analysis of microarrays (SAM) algorithm [28]. Differentially expressed genes were screened by using *t*-test and analysis of variance, if N is large number of our genes, it will generate a lot of false positives, then use controlling the FDR (false discovery rate) values corrected for multiple testing in the false-positive rate. Calculate the relative difference statistic d:

$$ \mathrm{d}=\frac{X_1^{\prime }-{X}_2^{\prime }}{s-{s}_0} $$

d, statistic measures the relative differences in gene expression are corrected d statistics. X1 ‘represents the average expression level of a state under genes, X2’ represents the average expression levels of gene, s represents the variance of the gene.

### Construction of co-expression network and module-mining

Construction of co-expression network mining based on the differentially expression value, weighted correlation network analysis (WGCNA) [29] was used for finding modules of correlated differential genes, summarizing such modules, relating modules to one another, and weighting module’s membership and contributed genes. All the genes used in WGCNA methods had been screened as previously described.

### Screening of differentially expressed module

Specific gene regulatory network module were screened in two conditions, and then determine the gene for each module in the two states within the overall expression differences, using a global analysis of variance method [30], Global-Ancova method based on correlation analysis of variance test set and a set of functional gene phenotype, P value tested with less than 0.05 network modules selected as differentially expressed module. The method may be R language Global-Ancova package implementation.

### Enrichment analysis of gene function

For a group identified gene sets, we used DAVID [31] tool - a software is based on the hyper geometric enrichment test methods of distribution test, to achieve function and the Kyoto Encyclopedia of Genes and Genomes (KEGG, http://www.genome.jp/kegg/) [32] pathway enrichment analysis.

### Survival analysis

By statistical analysis, we able to achieve some network modules consisted of differential selected genes with some chemotherapy related regulation factors. In view of these differential genes, we classified dataset GSE14764, GSE63885 and GSE49997 into subgroups, all the candidates are treated by chemotherapy. Prognosis analyses were conducted by SURVICAL package in R environment, and Kaplan-Meier estimates of overall survival (OS) respective 95 % confidence intervals (CIs) were provided for each cluster. In addition, for each dataset, Cox regression modeling [33] was used to control and assess for statistically significant prognostic factors, included adjustments for age, histology, and stage. Then the Pairwise comparisons between clusters were carried out in Cox model, based on calculated p-values, genes with p < 0.05 are considered to be relevant to the clinical characteristics and prognosis of ovarian cancer.

## Results

### Overall gene-expression profiling standardization

**Fig. 1:**
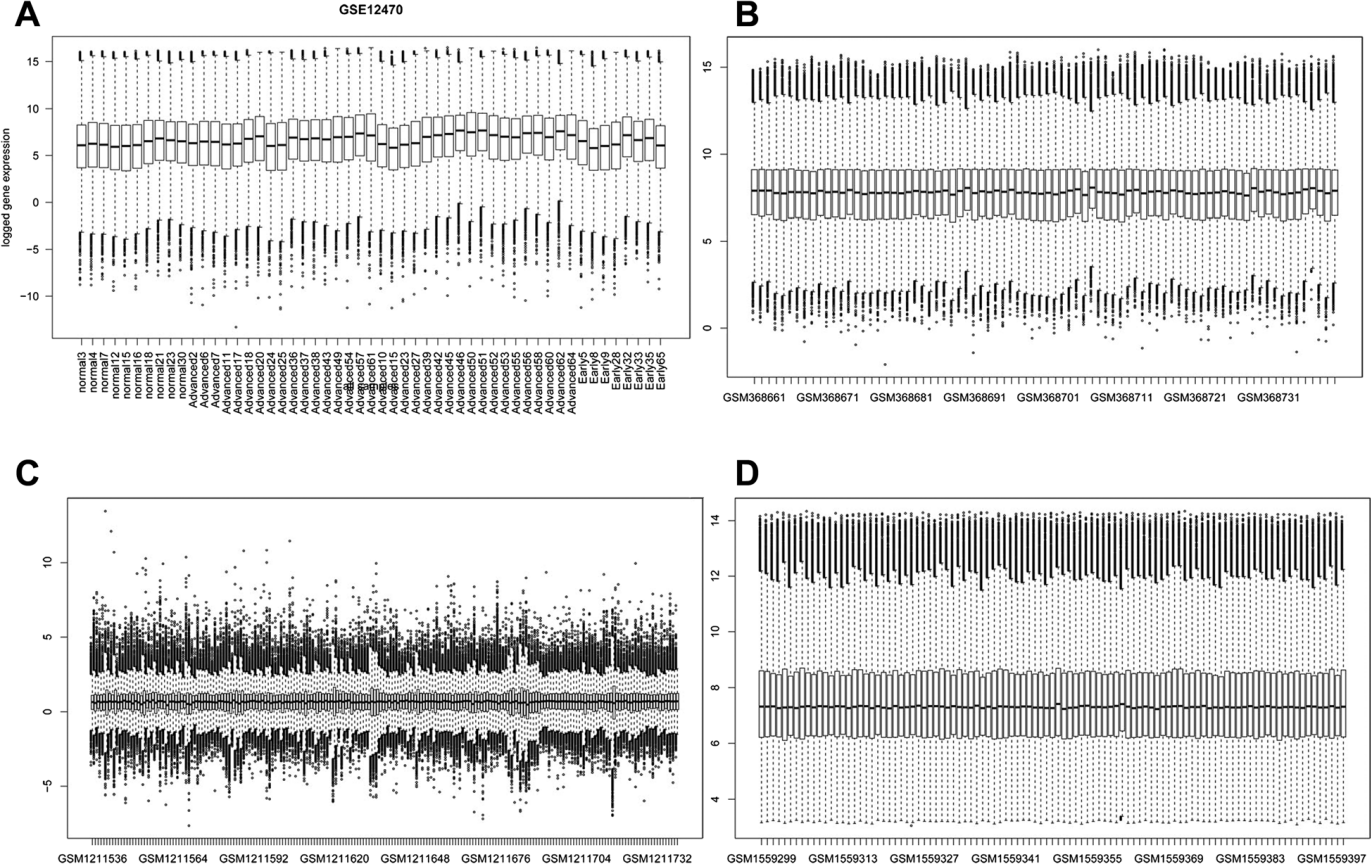
Box-plots of the distribution of gene expression values for analysis of ovarian cancer gene expression profiling (with a p-value of 0.05 and FC of 2.0). The abscissa represents each candidate IDs, while the vertical axis marks the data of genome expression of related patients, all the datein genome expression profiling are the mean value of many experiment locations. **a** GSE12470 dataset, 13356 genes in 53 samples (10 normal, 35 advanced, 8 early), (**b**) GSE14764dataset, 80 samples in different types of ovarian cancer and 13046 genes, C) GSE49997 dataset, 204 samples in various epithelial ovarian cancer and 16150 genes, D) GSE63885 dataset, 101 samples in different ovarian cancer and mRNA and 20693 genes. **a**, **b**, **c** are consisted of gene-expression data, while (**d**) reveals mRNA expression levels. Apparently, most genes’ expression values are approximately in each sample

As previously described, we generated original genome expression profiling and mRNA expression data got from each data set (GSE12470, GSE14764, GSE49997 and GSE63885), after using GEO database matching the probe ID in the platform to Gene Symbols, corresponding genes and gene’s IDs were collected from these data sets respectively. A quantitative genome expression distributions map are showed in type of box-plots (see Fig. 1), values from each dataset were linearized when provided as logarithms, raw files were converted into pre-processed data by RMA with default parameters [27].

### Ovarian cancer’s genetic screening and pathways analysis

According to the procedure adopted by Dai *et al.* [26], pre-processed data of 53 samples (Fig. 1a) were analyzed by SAM in R environment, samples including data from patients in various stage and non-cancer individuals. Lists of 3095 differentially expressed genes are collected (Accompanying Table 1), showing (i.e., fold change (FC) equals 2.0) were generated at SAM p-value thresholds of 5 %.

**Table 1:** Pathway analyses between normal person and patients in different stages (top 10)

Source	Name	p-value	q-value Bonferroni
REACTOME	Cell Cycle	3.03E-28	8.61E-25
REACTOME	Cell Cycle, Mitotic	7.62E-20	2.17E-16
REACTOME	Chromosome Maintenance	1.02E-19	2.91E-16
REACTOME	Telomere Maintenance	1.09E-19	3.11E-16
REACTOME	Deposition of New CENPA-containingNucleosomes at the Centromere	6.22E-17	1.77E-13
REACTOME	Nucleosome assembly	6.22E-17	1.77E-13
REACTOME	Meiotic Recombination	2.78E-16	7.90E-13
REACTOME	RNA Polymerase I Promoter Opening	3.36E-14	9.56E-11
REACTOME	RNA Polymerase I Transcription	8.11E-14	2.31E-10
REACTOME	RNA Polymerase I Chain Elongation	9.50E-14	2.70E-10

To identify the biological processes associated with these 3095 differential expressed genes, we explore the DAVID; http://david.abcc.ncifcrf.gov/). Compared with online human genome database, the top 10 enriched clusters with the 511 genes mainly distributed at cell cycle including mitosis, deposition of nucleosomes at the centromere, Chromosome Maintenance including Chromosome, telomere maintenance and nucleosome assembly, Regulation of RNA transcription level including RNA polymerase I (Table 1, Accompanying Table 2).

**Table 2:** Top 10 in weighted gene co-expression network analysis

Genes	Degree
RACGAP1	105.7018
UMPS	100.2887
NUSAP1	93.13226
RAD51AP1	92.99357
RAE1	92.71948
CBX3	90.26029
CENPL	89.92775
IARS	89.0546
MRPL3	89.03123
NEK2	87.25454

**Fig. 2:**
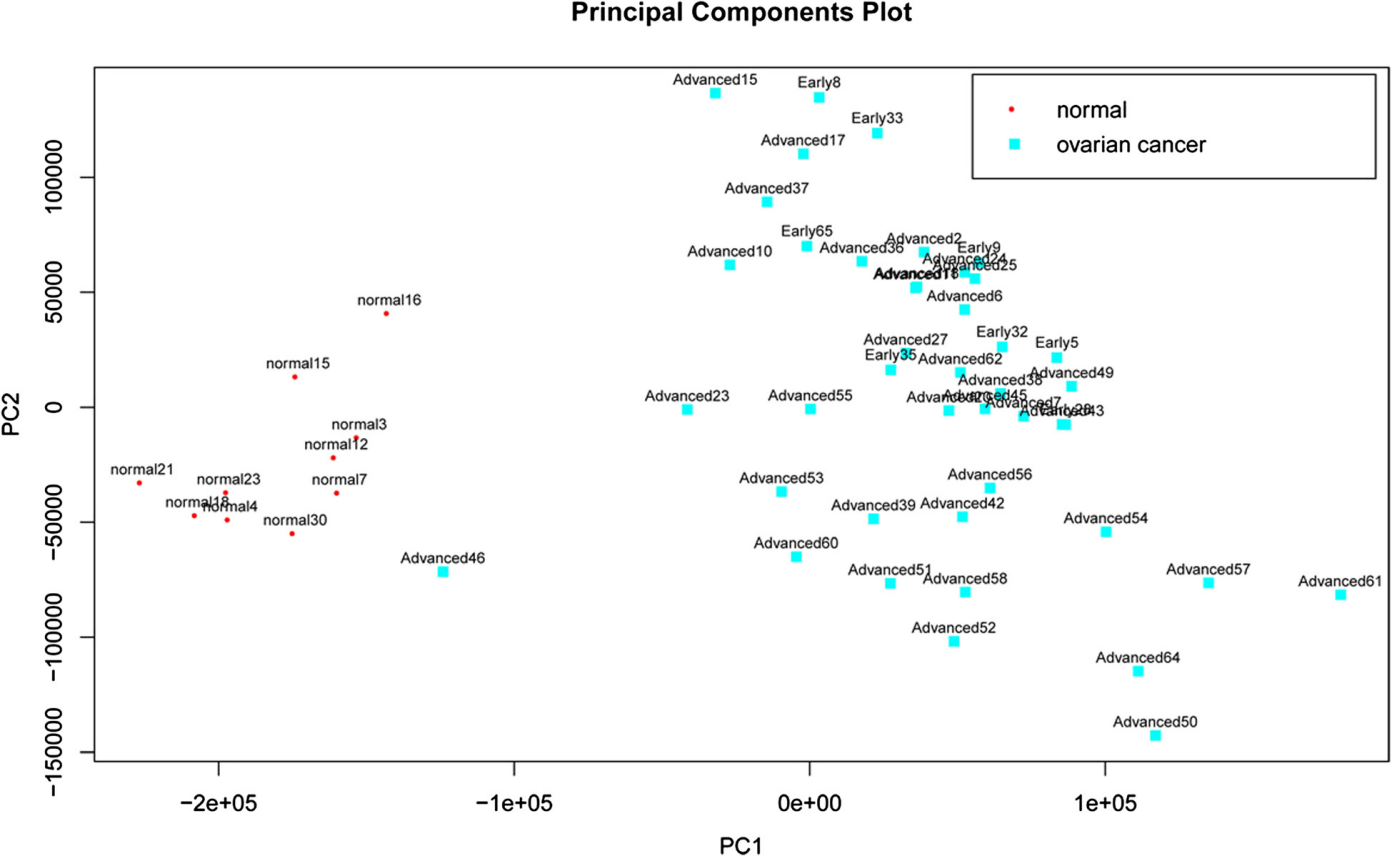
Clustering map base on 511 screened differential genes. Red spot indicate healthy individual, spot in blue indicate patient suffering from ovarian cancer, spots in different colors are effectively separated from each other

Based on these 511 genes related to top 10 pathways, overall 80 candidates were completely clustered by principal component analysis (PCA), which indicates a high-performance of differences genetic screening (Fig. 2).

### Differences genetic screening and pathways analysis on ovarian cancer in different stages

By using WGCNA software in R language, gene co-expression networks (Accompanying Table 3) are established from 3095 differential expression genes (Accompanying Table 1). Each gene was weighted and ranked by calculating the network edges, top 10 are showed in Table 2, Gene RACGAP1 [34], RAD51AP1 [35], RAE1 [36], NEK2 [37] had been reported as ovarian cancer related genes, while the others are newly defined related gene. In addition, these 3095 genes were divided into 17 modules (Table 3) by the block-wise, Modules function of WGCNA package. After further screening on Global-Ancova package using R language and comparing original gene-expression data set GSE12470, 4 network modules of differentially expressed cancer genes were identified (Table 4, details showed in Accompanying Table 4) as the representative module to apply function analyses because most of genes in the network are expressed in the candidate who suffered from cancer.

**Table 3:** Differential expressed gene divided into 17 networkmodules

Module_name	F.value	Gene_num	p.approx
Black	0.775321	120	0.32445
Blue	1.993077	546	0.047693
Brown	0.822257	294	0.487956
Cyan	1.898666	24	0.104177
Green	1.501158	200	0.122757
Greenyellow	3.187852	32	0.01002
Grey	1.6997	464	8.81E-06
Lightcyan	2.393375	22	0.066907
Magenta	1.248493	60	0.215386
Midnightblue	0.453455	23	0.710623
Pink	1.184971	75	0.217628
Purple	1.265749	55	0.215887
Red	1.639867	161	0.051571
Salmon	1.295175	31	0.201977
Tan	2.495361	31	0.0358
Turquoise	1.755095	717	0.073338
Yellow	1.025866	240	0.30132

**Table 4:** Pathway analyses in dominantnetwork modules composed by differential expression ovarian cancer genes

Module	Category	Term	P-value
Blue	KEGG_PATHWAY	hsa00150:Androgen and estrogen metabolism	7.72E-07
Blue	KEGG_PATHWAY	hsa00970:Aminoacyl-tRNA biosynthesis	0.001102
Blue	KEGG_PATHWAY	hsa00140:Steroid hormone biosynthesis	0.002036
Blue	KEGG_PATHWAY	hsa00860:Porphyrin and chlorophyll metabolism	0.00247
Blue	REACTOME_PATHWAY	REACT_1698:Metablism of nucleotides	0.009412
Blue	KEGG_PATHWAY	hsa00983:Drug metabolism	0.036080937
Greenyellow	KEGG_PATHWAY	hsa03320:PPAR signaling pathway	0.053198
Greenyellow	REACTOME_PATHWAY	REACT_602:Metabolism of lipids and lipoproteins	0.086351
Grey	KEGG_PATHWAY	hsa04916:Melanogenesis	0.006757
Grey	REACTOME_PATHWAY	REACT_17044:Muscle contraction	0.015831
Grey	REACTOME_PATHWAY	REACT_13:Metabolism of amino acids	0.021384
Grey	BIOCARTA	h_ghrelinPathway:Ghrelin: Regulation of Food Intake and Energy Homeostasis	0.028498
Grey	KEGG_PATHWAY	hsa00512:O-Glycan biosynthesis	0.029358
Tan	KEGG_PATHWAY	hsa00500:Starch and sucrose metabolism	0.001807
Tan	REACTOME_PATHWAY	REACT_474:Metabolism of carbohydrates	0.002231

GO and KEGG analysis on these 4 modules (Table 4) shows blue modules is mainly take part in female metabolism regulation and controlling: Androgen and estrogen metabolism and Steroid hormone biosynthesis which straightly related to ovarian functions, Aminoacyl-tRNA biosynthesis which play a key role in protein synthesis [38] and has been suggested to be associated with the progression of various ovarian cancers [39, 40], most interested is porphyrin and chlorophyll metabolism pathways also be involved into ovarian cancer progression, porphyrin was reported as treatment elements for ovarian cancer [41], while chlorophyll as important grapevine iron nutrition for blood [42, 43] which most females are short for it [44], besides, some reporter illustrated cancer resistance protein can against the porphyrin and chlorophyll metabolism [45], thus, blue module may potentially denotes the progress of ovarian cancer and support to our subsequence prognosis analysis. Besides, gene UMPS ranked second was involved in pathway of aminoacyl-tRNA biosynthesis, further suggests that UMPS could be related to a certain ovarian cancer. And gene IARS belongs to drug metabolism pathway in blue module ranked eighth in Table 2, suggesting that this gene maybe important for applicability of drug treatment in specific case.

Greenyellow module is mainly related to PPAR signaling pathway, which is involved in ovarian follicle development [46] and ovarian cancers progress [47]. Grey module is mainly devoted to melanogenesis. Presently, no representation shows melanogenesis is related to cancer progression, but melanogenesis is regarded as a potential instruction for understanding of complex diseases [48]. In currently study, we select the modules to evaluate ovarian cancer in different stages and various types, thus, this module probably take an important part in subsequence prognosis analysis for patients in various conditions, beside, the other functions of this modules also help to analysis cancer proceeding like amino acids metabolism and energy homeostasis. Tan module is mainly devoted to carbohydrates and sucrose metabolism, and this is a risk factor for many cancer [49] and female ovarian health [50], also very important to diagnosis of advanced ovarian cancer patients [51, 52].

All these supported researches and relevance data illustrated that we had generated network modules from differential expression genes of various ovarian cancers successfully, and these networks are competent for predict ovarian cancer’ subgroups, also potentially indicate the proceeding of ovarian cancer in different patients.

### Prognostic analysis of subgroups of ovarian cancers

**Fig. 3:**
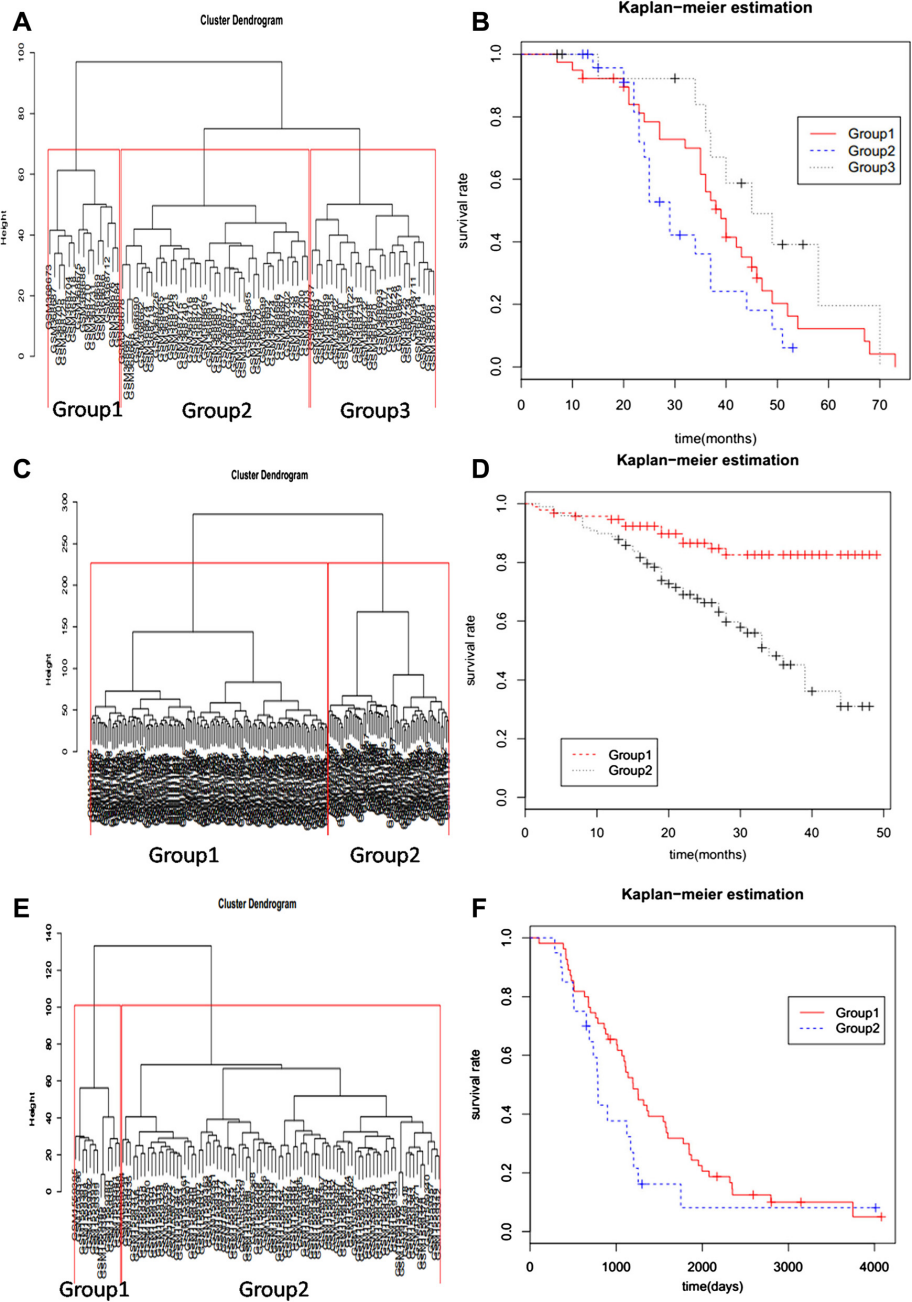
Cluster analysis: Heat map profiles of ovarian cancer patients with 1073 extracted differential genes from GSE12470 data set (n = 53). **a** Heat map profiles of extracted differentiated genes and various ovarian cancer patients from GSE14764 dataset (genome expression, n = 80), the Kaplan-Meier curves are with respect to (**b**) overall survival (OS) rite at non-significant P = 0.0323, (**c**) Heat map profiles of extracted differentiated genes and various ovarian cancer patients from GSE49997 dataset (mRNA expression, n = 204), corresponding Kaplan-Meier curves (**d**) with a non-significant P = 1.02e - 05, (**e**) Heat map profiles of extracted differentiated genes and various subtypes of epithelial ovarian cancer patients from GSE63885 dataset (genome expression, n = 101), the Kaplan-Meier curves are with respect to (**f**) overall survival (OS) rite at non-significant P =0.0781,A) is for prognosis trials, (**c, e**) are used to verify the availability of selected modules and extracted differential expression genes. All estimates of OS respective 95 % confidence intervals (Cis)

1073 differential expression genes involved in the 4 dominant network modules were generated from GSE12470 expression dataset as previous described. By using SUVIVLE package in R basing on these differential genes, GSE14764 dataset composed by various ovarian cancer patients’ gene expression profiles (n = 80) were classified into 3 subgroups (Fig. 3a). Pair wise comparisons between clusters based on p-values were carried out by Kaplan-Meier estimates of OS respective 95 % confidence intervals (CIs). Kaplan-Meier estimates of Fig. 3a has been showed in Fig. 3b with P = 0.0323.

In order to verify the availability of the prognostic functions of these 4 modules, we useGSE49997 and GSE63885 datasets to repeat the same experiment. GSE49997 [23] is composed by mRNA expression data from epithelial ovarian cancer patients (n = 204) while GSE63885 datasets are consisted by genome expression data from various ovarian cancers. According to the original articles, candidates in GSE49997 [23] dataset are classified into the clinic-pathologic parameters of the histological serous and non-serous tumor subtypes, each subtypes can be divided into 2 subclasses derived from International Federation of Gynecology and Obstetrics stage-directed supervised classification approach (IFGO). One group’s (subclass2) conditions deteriorated extremely from a certain time point and appear much lower livability in both serous and non-serous histological subtypes than another (subclass1)’s, as revealed by univariate analysis (hazard ratios [HR] of 3.17 and 17.11, respectively; P 0.001) and in models corrected for relevant clinic pathologic parameters (HR 2.87 and 12.42, respectively; P 0.023). Similarly, candidates in GSE63885 [22] datasets adapt the same classification approach(IFGO), and they discovered that histological type could be a confusing factor and gene expression exploration of ovarian carcinomas should be performed on histologically homogeneous groups to direct the prognostic analysis on chemotherapy. In their experiment, clinical endpoints like overall survival, disease-free survival, tumor response to chemotherapy are not confirmed by validation either on the same group or on the independent group of patients, just CLASP1 gene with BRCA1 mutation status related to one ovarian cancer subclass which tend to deteriorate easily.

Comparatively, heat map profiles in current researches (Fig. 3c and Fig. 3e showed) showed the samples from GSE49997 and GSE63885 dataset had been efficiently divided into 2 groups base on the same differential expressed genes and 4 network modules used in Fig. 3a, which are identical with the original dataset information. In Kaplan-Meier estimates of OS respective 95 % confidence intervals (CIs) were provided for these two heat maps with p equals to 1.02e-05 and p equals to 0.0781 respectively. According to these two verification models and similarities in classification to original data sources we described above, the selected 1073 different genes in 4 majority network modules is competent to classify ovarian cancer into subtypes that are prognostic of different chemotherapy outcome, especially for epithelial ovarian cancer and ovarian germ cell cancer (especially for stage 4 and stage 5), which are notorious for diagnosis and distinction at the early stage with analogous morphological characteristics. In addition, the modules we established may prefer much more accuracy and practicability, as GSE63885 [22] datasets with less stringent criteria for gene selection (FDR <10%and uncorrected p-value <0.001).

**Fig. 4:**
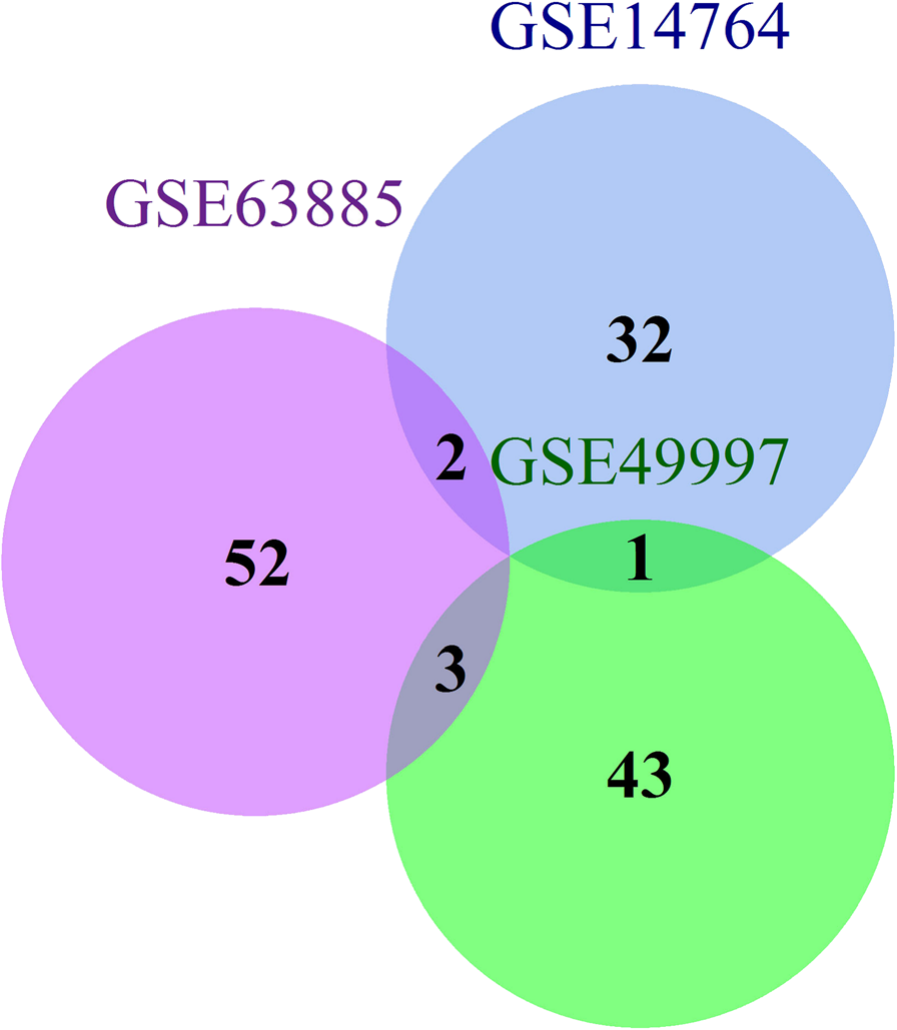
Three data sets COX univariate regression analysis were screened for ovarian cancer prognostic gene Venn diagram

For further extraction and prognosis of genes directly related to ovarian cancer survival, we used univariate COX regression method to calculate the correlation between genes and survival prognosis within the module, GSB14764 dataset genes associated with prognosis in a total of 35 genes; GSE49997 dataset and prognosis related genes, a total of 47 genes (Additional file 1: Table S5); GSE63885 dataset and prognosis with a total area of Venn diagram with 57 genes (Additional file 1: Table S6). View these three ovarian cancer prognostic gene intersection situations, find the intersection between any two relatively small (Fig. 4), the intersection of the six genes LRRC8D, TTC304, TFCP2L1, LIBRINEPOR, PAR52. Outstandingly, dysregulation of this EPOR may affect the growth of certain tumors [53, 54].

## Discussion

As previously described, ovarian cancer like epithelial ovarian cancer and ovarian germ cell cancer has difference subclasses, but it is hard to distinguish the malignant from carcinoid tumors due to the multitude of clinical and history pathological aspects [8], lack of precursor lesions [9] and their evolution [10], which cause the bad one with a low survival rate and complicated due to frequent development of resistance to standard therapies and asymptomatic nature of the early stage. Thus, recently, more and more researches are focus on genome level analysis aim at recognize collaborative gene and relatively network modules, which will bring out some newly efficiently diagnoses, and help to the cancer prevent and treatment to individuals base on targeted chemotherapy.

Current established genetic ovarian carcinomas prognostic pattern contains 1073 difference expression genes involved in the 4 dominant network modules successfully divided a dataset with random OCA cases (n = 80) into 3 groups (p = 0.0323, 95 % CIs in Kaplan-Meier). Two other previously reported datasets verified this classification is available and can be used in both genome (n = 204, p =1.02e-05, 95 % CIs in Kaplan-Meier) and mRNA (n = 101, p =0.0781, 95 % CIs in Kaplan-Meier) profiles, also demonstrated that this pattern can be used to distinguish epithelial ovarian cancer and ovarian germ cell cancer subclasses that trend todevelopmalignantly.6 prognosis related genes were selected by COX regression analysis (LRRC8D, TTC30A, TFCP2L1, LMBR1, EPOR and PARS2), these difference genes regulate modules through the whole work, rather than a few genes play a prognostic classification, which can make the outcome much more convincing. Beyond them, EPOR is famous for its affection to tumor growth [53, 54], support the function to divide the malignant epithelial ovarian cancer or ovarian germ cell cancer from carcinoid tumors;TTC30A and LRRC8D are rarely reported before, but recent statistics shows that these two gene related to immune system, and may have regulation ability to host protein [55–57], these can be considered in chemotherapy methods choosing. In addition, corresponding to earlier pathway analysis (Aminoacyl-tRNA biosynthesis in blue module, Table 4), PARS2 encodes a putative member of the class II family of aminoacyl-tRNA synthetases, further suggested a highly correlated gene networks in currently generated modules. What is importantly is that TFCP2L1 probably contribute to the differentiation of cancer stem cells, as embryonic stem cell self renewal pathways converge on the transcription factor Tfcp2l1 [58], and this never been reported before.

The present study describes a validation analysis of a previously defined gene signature to establish its relevance as a clinically useful prognostic factor. While the accuracy of prognostic outcome restricted by two elements, the routine use of recently published new prognostic factors in clinical practice has had limited success, and the updated gene databases.

## Additional file

Additional file 1:
**Tables S5 and S6.**
