# Supplementary material for: Gene expression profiling of ovarian carcinomas and prognostic analysis of outcome
Source: J Ovarian Res. 2015 Jul 31;8:50. doi: 10.1186/s13048-015-0176-9 (PMC4521463; doi:10.1186/s13048-015-0176-9)
Supplement: Additional file 1: — Tables S5 and S6. [file 13048_2015_176_MOESM1_ESM.docx]

**Supplementary table 5**

| GeneSymbol | P_value | coef |
| --- | --- | --- |
| COL10A1 | 0.000955975 | 0.224848536 |
| RPP38 | 0.001249675 | -0.703111459 |
| CYP26A1 | 0.001301374 | 0.336813781 |
| WNT6 | 0.002025534 | -0.306765057 |
| KCNJ5 | 0.002275394 | 0.470188242 |
| SPINK1 | 0.002486898 | 0.201879378 |
| COL9A1 | 0.003205096 | -0.192586641 |
| PARS2 | 0.003278766 | -0.463640362 |
| PKP2 | 0.003493989 | -0.31555252 |
| LMBR1 | 0.003943666 | -0.549454647 |
| NARS2 | 0.004860052 | -0.485551065 |
| MMP11 | 0.00494301 | 0.171614698 |
| GJB2 | 0.006347078 | 0.28806642 |
| ABCB9 | 0.007120334 | -0.331230071 |
| SST | 0.007652876 | -0.18110259 |
| RPUSD2 | 0.007757915 | -0.44658047 |
| C1orf109 | 0.008825475 | -0.328534243 |
| LRRC8D | 0.009549053 | -0.344991677 |
| FAT2 | 0.011438864 | 0.346606472 |
| DDX6 | 0.012433764 | -0.396292105 |
| NETO2 | 0.013106387 | 0.309265983 |
| EPOR | 0.013304447 | 0.272482391 |
| ADSSL1 | 0.015302523 | 0.411721766 |
| ARSD | 0.015986202 | -0.499039934 |
| RPL39 | 0.017058031 | -0.819905771 |
| PRAME | 0.017317493 | -0.122198876 |
| LRRC61 | 0.017738616 | -0.399954624 |
| JTB | 0.017909293 | -0.355817022 |
| BTC | 0.018283572 | -0.333052391 |
| ANKH | 0.018955222 | 0.495464612 |
| FOXA2 | 0.021721887 | -0.473242082 |
| C14orf142 | 0.022766137 | -0.329457817 |
| ECHDC3 | 0.024863429 | -0.273301554 |
| TMEM81 | 0.026171812 | -0.385830035 |
| GDF15 | 0.026747482 | 0.22567914 |
| TYSND1 | 0.027712776 | -0.257930514 |
| TRIT1 | 0.028801276 | -0.376757605 |
| PRMT6 | 0.031409396 | -0.316386229 |
| AASDHPPT | 0.034056871 | -0.296080424 |
| XAGE5 | 0.035116404 | -0.32624575 |
| HMGN3 | 0.037481806 | -0.438434091 |
| KATNB1 | 0.038432638 | -0.297922441 |
| CTPS2 | 0.038583436 | -0.290532234 |
| CYP4B1 | 0.044253923 | -0.138191225 |
| KLK2 | 0.045325144 | 0.328138353 |
| AGBL2 | 0.046316705 | -0.485136369 |
| ALG6 | 0.046538364 | -0.358931101 |

**Supplementary table 6**

| GeneSymbol | P_value | coef |
| --- | --- | --- |
| YIPF3 | 0.000194769 | -1.42376 |
| FDFT1 | 0.000332164 | -0.95425 |
| TMED10 | 0.000759423 | -1.69462 |
| CARHSP1 | 0.001999532 | 1.76595 |
| TARBP1 | 0.002747949 | -0.85215 |
| DBNDD1 | 0.003296708 | 0.853199 |
| C6orf130 | 0.003412052 | -0.80362 |
| TSFM | 0.005919991 | -0.93393 |
| LIPH | 0.006326581 | 0.670958 |
| MVK | 0.007371152 | 1.924068 |
| SLC27A6 | 0.009571541 | -0.26067 |
| LRRC1 | 0.010084388 | -1.26172 |
| INPPL1 | 0.010932644 | 0.814588 |
| TFCP2L1 | 0.012342458 | 0.500172 |
| SMUG1 | 0.015273031 | -1.11648 |
| ZNF202 | 0.015450725 | -1.07147 |
| TTC30A | 0.015510742 | -0.39806 |
| UNC5CL | 0.015576225 | -0.77085 |
| SEC23B | 0.015625527 | -0.73871 |
| OCLN | 0.015635147 | 0.572642 |
| C14orf104 | 0.015734612 | -0.48084 |
| CSRP2BP | 0.015828882 | -1.32505 |
| COX18 | 0.015970563 | -1.0835 |
| GMPR | 0.016138871 | -0.29425 |
| STK31 | 0.017410917 | 0.44558 |
| ZNF625 | 0.0184052 | -1.28863 |
| SHMT2 | 0.019003987 | -0.51522 |
| ST3GAL6 | 0.020593453 | -0.4786 |
| MGAT4B | 0.021757437 | 0.465879 |
| AMY2B | 0.023061505 | -0.17894 |
| DKFZp761E198 | 0.024672563 | 0.692309 |
| ZNF646 | 0.025124969 | -1.19947 |
| EPB41 | 0.025998601 | 1.181973 |
| NFX1 | 0.027225353 | -0.76822 |
| CD22 | 0.027355762 | -0.80122 |
| LMBR1 | 0.027614271 | -0.5168 |
| C1orf156 | 0.027792543 | -0.55075 |
| LYPLA1 | 0.027951144 | 0.631896 |
| NUDT6 | 0.032592361 | -0.53398 |
| CTSB | 0.033805338 | -0.48939 |
| PCCB | 0.033909543 | 0.885929 |
| SLC33A1 | 0.035541124 | -0.61166 |
| XRCC6BP1 | 0.035982235 | -0.87936 |
| SPINK5 | 0.036409549 | 0.538659 |
| LMO7 | 0.036795658 | 0.850131 |
| IL4I1 | 0.037227953 | -0.2465 |
| ACP6 | 0.039934736 | -0.41046 |
| GTF3C2 | 0.040293732 | 0.782378 |
| MYBPH | 0.041405614 | 1.276463 |
| ENOSF1 | 0.042200193 | -0.45298 |
| PVALB | 0.042611367 | 0.342538 |
| EPOR | 0.042858608 | -0.49058 |
| TSPAN3 | 0.043952336 | -0.87452 |
| C11orf1 | 0.044009467 | -0.68927 |
| PARS2 | 0.045182466 | -0.65724 |
| CCDC6 | 0.046997535 | 0.426605 |
| MLLT6 | 0.049753421 | -0.91559 |
